# A Space to Promote Intentional Thoughtful Action

**DOI:** 10.1089/heq.2016.29001.edi

**Published:** 2017-01-01

**Authors:** Ana Núñez, Jordan L. Schilling

**Affiliations:** ^1^ Office of Diversity, Equity & Inclusion, Drexel University College of Medicine, Philadelphia, Pennsylvania.; ^2^ Mary Ann Liebert, Inc., New Rochelle, New York.

In healthcare, we strive to have excellent care, good outcomes, and healthy communities. But as we know, the “playing field” is not level for all the players. Achieving health equity for vulnerable populations, and eliminating health disparities, is an even more critical issue especially now in the United States. Imagine if we would be unable to craft a Healthy People 2030 because we had no disparities? We are a long way away from that dream. *Healthy People 2020* continues to highlight that vulnerable populations experience greater obstacles to health based on their religion, racial or ethnic group, socioeconomic status, gender, sexual orientation or gender indemnity, mental health, disabilities, geographic location, or other characteristics historically linked to discrimination or exclusion.^†^ There is a critical need for an authoritative resource for organizations and individuals who serve these populations at the community, state, regional, tribal, and national levels. Similarly, there is a pressing need to disrupt silos of information so that the innovations and trends can be accessed by readers, researchers, and thinkers across disciplines and specialties who want be energized by a breadth of ideas and interventions.

*Health Equity* was founded to deliver authoritative, peer-reviewed, information and contribute to the empowerment of communities to identify and solve pervasive problems related to health and wellness. The Journal will be a critical and timely source of information for physicians, nurses, and other healthcare providers; policy makers; psychologists and social workers; educators; and researchers; among others. *Health Equity* will publish a wide range of articles on translational research to prevention, diagnosis, treatment, and management of disease and illness toward the goal of optimal outcomes and ultimately health equity for all. We seek to stimulate a national conversation about what works and what needs to be done. The Journal's open access format will allow for a collaborative, inter and transdisciplinary space to address practical interventions that will ultimately enhance the lives of patients and collectively aid in the discourse promoting health equity.

*Health Equity* is supported by an outstanding team of Associate Editors, including Thanakorn Jirasevijinda, MD, NewYork-Presbyterian Hospital/Weill Cornell Medical Center; Mary Kimmel, MD, University of North Carolina; Charles P. Mouton, MD, Meharry Medical College; Francisco Garcia, MD, Arizona Department of Health; and Michelle L. Rogers, PhD, Drexel University; and an expansive editorial board of leaders in health disparities research and practice.

We are thankful to have support from the W.K. Kellogg Foundation. Its support will ensure that the Journal is accessible as widely as possible and provides a framework for achieving health equity for children, families, and communities. We are excited to launch the Journal and create a space for health equity to grow. Lastly, many thanks go to the publisher and team at Mary Ann Liebert, Inc., for making this dream possible. Please join us in the work and the conversation!

## Notes

^†^ Healthy People 2020. www.healthypeople.gov

